# Development of diabetes mellitus after hematopoietic stem cell transplantation for childhood leukemia

**DOI:** 10.1186/1687-9856-2015-S1-P28

**Published:** 2015-04-28

**Authors:** In Ah Jung, Yeon Jin Jeon, Won Kyoung Cho, Jae Wook Lee, Nak Gyun Chung, Min Ho Jung, Bin Cho, Byung Kyu Suh

**Affiliations:** 1 Department of Pediatrics, College of Medicine, The Catholic University of Korea, Seoul, Korea

## Aims

We investigated clinical features of newly diagnosed diabetes mellitus (DM) after hematopoietic stem cell transplantation (HSCT) for treatment of childhood leukemia.

## Methods

Between April 2009 and March 2014, total 124 patients (73 males, 51 females) were visited the clinic of pediatric endocrinology for routine follow-up check after HSCT for leukemia. Among them, five patients developed DM (4 males, 1 female). We retrospectively reviewed medical charts including laboratory findings.

## Results

Three patients were diagnosed as acute lymphoblastic leukemia who received total body irradiation and chemotherapy. The other two patients were diagnosed as acute myeloblastic leukemia. The mean age at HSCT was 8.0 ± 4.2 years. Four out of five patients developed chronic graft-versus-host disease (GVHD) and treated with steroid more than 2 years. The mean age at diagnosis of DM was 15.8 ± 1.8 years and the time interval between HSCT and DM was 7.8 ± 4.4 years. Three patients showed obesity depend on body mass index (>95^th^ percentile for sex and age). No one showed antibodies related with pancreatic β-cell. All five patients showed hyperinsulinemia with mean fasting insulin levels at diagnosis of DM was 13.3 ± 9.2 μIU/mL. The mean homeostasis model assessment of insulin resistance index (HOMA-IR) of patients was 4.39 ± 2.01.

## Conclusion

GVHD, long-term steroid treatment and insulin resistance seem to be close related to develop of DM after HSCT for treatment of childhood leukemia.

